# Changing behavior – ensuring hand hygiene is an institutional priority

**DOI:** 10.1186/1753-6561-5-S6-P111

**Published:** 2011-06-29

**Authors:** J Bradford, J Brett, A Bull, B Kennedy, S Borrell, A McMillan, M Richards

**Affiliations:** 1 VICNISS Coordinating Centre, Melbourne, Australia; 2 Quality, Safety and Patient Experience, Department of Health, Victoria, Australia

## Introduction / objectives

In 2004 the Victorian Quality Council developed a state-wide model aiming to improve hand hygiene and reduce healthcare associated infections across Victoria. Many aspects of this culture change program have been incorporated into the National Hand Hygiene Initiative which commenced in 2008.

## Methods

All 86 acute public Health Services in Victoria were funded to participate and submit three hand hygiene compliance audits per year. Education, resources and auditor training was provided. Healthcare worker hand hygiene compliance was assessed by direct observation using a tool based on the WHO 5 Moments.

In 2009 an organisational hand hygiene compliance benchmark was included in the Health Service Performance Management Framework of the Department of Health. Health Service Chief Executive Officers (CEO) were provided quarterly feedback as to their performance against the agreed benchmark, this performance was also made known between organisations.

## Results

Health Services have progressively improved compliance and most recently 95% achieved the current benchmark of 65%. Feedback from Infection Control Consultants has also been positive, reporting a marked increase in executive support for the hand hygiene initiative.

## Conclusion

The successful implementation and sustainability of any hand hygiene initiative requires leadership from all levels including government, health service CEO, and ownership of the program by individual clinical areas and clinicians. Hand hygiene compliance as a performance indicator promotes executive commitment to the program and ensures hand hygiene is an institutional priority.

## Disclosure of interest

None declared.

